# Cytotoxicity and Antioxidant Activity of 23 Plant Species of Leguminosae Family

**Published:** 2012

**Authors:** Farahnaz Khalighi-Sigaroodi, Maryam Ahvazi, Abbas Hadjiakhoondi, Mitra Taghizadeh, Darab Yazdani, Shahram Khalighi-Sigaroodi, Siamak Bidel

**Affiliations:** a Department of Pharmacognosy and Pharmaceutics, Institute of Medicinal Plants, Iranian Academic Center of Education, Culture and Research, ACECR, Karaj, Iran.; b Department of Herbarium, Institute of Medicinal Plants, ACECR, Karaj, Iran.; c Department of Pharmacognosy and Medicinal Plants Research Center, Faculty of Pharmacy, Tehran University of Medical Sciences, Tehran, Iran.; d Natural Resources Faculty, University of Tehran, Karaj, Iran.; e Department of Health Promotion and Chronic Disease Prevention, National Institute of Health and Welfare, Helsinki, Finland.; f Department of Public Health, Faculty of Medicine, University of Helsinki, Helsinki, Finland.

**Keywords:** Cytotoxicity, Antioxidant, Leguminosae, *Taverniera spartea*

## Abstract

Numerous studies have been focused on natural anticarcinogenic agents. Many antioxidants have been identified as anticarcinogens. Antimutagens have also been proposed as cancer chemopreventive agents. The use of natural products as anticancer has a long history that began with traditional medicine. The aim of this study was to evaluate cytotoxicity and antioxidant activity of twenty-three plant species of Leguminosae family from different regions of Iran.

Twenty-three plant species of Leguminosae family were collected in May-June 2009 from different regions of Iran.Methanol extracts of these species were tested through the brine shrimp lethality assay in order to detect potential sources of novel cytotoxic compounds. The total antioxidant activity was evaluated with DPPH free radical-scavenging method.

The extracts of twelve species showed moderate cytotoxicity against brine shrimp (LC50 between 30 and 50 μg/mL). The extracts of Taverniera spartea and Tephrosia persica showed significant cytotoxicity (LC50 < 30 μg/mL) with LC50 values of 0.34 and 2.43 μg/mL, respectively, whereas the positive control, thymol showed a LC50 value of 1.37 μg/mL. The chloroform fractions of the latter two species were subjected to the brine shrimp lethality assay with LC50 values of 113.79 and 1.23 μg/mL, respectively. In comparing antioxidant capacities, Gleditschia caspica and Taverniera spartea showed significant antioxidant activity (IC50 < 50 μg/mL) with LC50 values of 14.54 and 20.32 μg/mL, respectively.

It could be seen among 23 tested plant species that Taverniera spartea had the most cytotoxic and antioxidant activity and was the best candidate for these effects. Further investigations are necessary for chemical characterization of the active compounds and more comprehensive biological assays.

## Introduction

Numerous studies have been focused on natural anticarcinogenic agents. Many antioxidants have been identified as anticarcinogens. Antimutagens have also been proposed as cancer chemopreventive agents. Therefore, the regular consumption of antimutagens can reduce genotoxic effects of mutagenic and carcinogenic factors (1, 2).

The use of natural products as anticancer has a long history that began with traditional medicine. Several drugs in chemotherapy were isolated from plant species or derived from a natural prototype. They include vinblastine, vincristine, taxanes, etoposide and teniposide, the semisynthetic derivatives of epipodophyllotoxin, camptothecin, irinotecan, and topotecan and several others. Over 50% of the drugs in clinical trials for anticancer activity were isolated from natural sources or were related to them (3).

The brine shrimp lethality assay is a general bioassay that seems to be capable of detecting a wide spectrum of bioactivity present in crude extracts. The commercial availability of inexpensive brine shrimp eggs, the low cost, the safety and ease of performing the assay, as well as no special technology requirement make this a very helpful bench-top tool for the phytochemistry laboratory. The lethality to brine shrimp is recommended as an effective prescreen to existing cytotoxicity and antitumor assays. A number of studies have established the use of the brine shrimp assay to screen plants commonly used as pesticides, anticancer, and with molluscicidal, larvicidal, fungicidal, and cytotoxic activity. Lastly, this assay has been used successfully to biomonitor the isolation of cytotoxic, antineoplastic, antimalarial, insecticidal, and antifeedant compounds from plants (4).

Antioxidants are usually added to foods to prevent the radical chain reactions of oxidation. They act through inhibiting the initiation and generation step leading to the termination of reaction and delay the oxidation process. However, synthetic antioxidants such as butylated hydroxyanisole and butylated hydroxy toluene are restricted because of their potential for toxic and carcinogenic effects. Therefore, there has been a significant interest to find natural antioxidants to replace the synthetic compounds in food applications and a growing tendency in consumer preferences for natural antioxidants, all of which has given more impetus to explore the natural sources of antioxidants (1).

The present study aims to provide data on the cytotoxic potential of 23 plant species of Leguminosae family from different regions of Iran on developing brine shrimp nauplii. The antioxidant activity of the species with potent cytotoxicity effects have been evaluated using the spectrophotometric 1,1-diphenyl-2-picrylhydrazyl (DPPH) free radical-scavenging method.

## Experimental

*Plant material*

Twenty-three plant species of Leguminosae family were collected in May-June 2009 from different regions of Iran. The plant species were identified and voucher specimens have been deposited at Institute of Medicinal Plants Herbarium (IMPH) (Table 1). The plant parts were air-dried under the shade and ground using a laboratory mill and a kitchen blender.

**Table 1:** List of plant species collected from different regions of Iran and yields of the methanol extracts

**No**	**Plant species voucher no.**	**Time of collection**	**Area of collection**	**Part used**	**Yield (%w/w)**
1	*Astragalus squarrosus *Bunge (IMPH^a^ 611)	May 2009	The road of Quhin to Loshan, Qazvin province	Aerial parts	24
2	*Caesalpinia gilliesii *(Hook.) D.Dietr. (IMPH 610)	May 2009	Chabahar, Sistan and Baloochestan province	Aerial parts	12
3	*Crotalaria burhia *Buch.-Ham. ex Benth. (IMPH 612)	May 2009	The road of Chabahar to Baris, Sistan and Baloochestan province	Stems	10
4	*Gleditschia caspica *Desf. (IMPH 613)	May 2009	Chaparpardzaman, The road of Zibakenar to Golshan, Gilan province	Leaves, Fruits	32
5	*Glycyrrhiza glabra *L. var. *glabra *L. (IMPH 614)	June 2009	The road of Moshkan to Kashan, Esfahan province	Aerial parts	34
6	*Glycyrrhiza glabra *L. var. *glandulifera *(Waldst. and Kit.) Boiss. (IMPH 615)	June 2009	The road of Esafahan to Naiin, Esfahan province	Aerial parts	24.5
7	*Indigofera articulata *Gouan. (IMPH 616)	May 2009	Tis, The road of Chabahar to Baris, Sistan and Baloochestan province	Aerial parts	20
8	*Indigofera intricata *Boiss. (IMPH 617)	June 2009	Bandar Abbas, Hormozgan province	Aerial parts	13.5
9	*Lathyrus annus *L. (IMPH 618)	May 2009	Galangoodeh, Bandar Anzali, Gilan province	Aerial parts	18.5
10	*Lathyrus sativus *var. *stenophyllus *Boiss. (IMPH 619)	May 2009	Galangoodeh, Bandar Anzali, Gilan province	Aerial parts	30
11	*Lotus corniculatus *L. subsp. *corniculatus *(IMPH 620)	May 2009	Bandar Anzali, Gilan province	Aerial parts	16.9
12	*Medicago rigidula *(L.) All. (IMPH 621)	May 2009	Hashtgerd, Tehran province	Leaves, Fruits	13.5
13	*Melilotus indicus *(L.) All. (IMPH 622)	May 2009	Talesh mahalleh, Gilan province	Aerial parts, Roots	19
14	*Onobrychis altissima *Grossh. (IMPH 623)	May 2009	Mehrshahr, Tehran province	Aerial parts	17.8
15	*Sophora alopecuroides *L. (IMPH 624)	June 2009	The road of Natanz to Moorcheh khort, Esfahan province	Aerial parts	29
16	*Sophora pachycarpa *C.A. Mey. (IMPH 625)	June 2009	Natanz, Esfahan province	Aerial parts	22.5
17	*Taverniera cuneifolia *Arn. (IMPH 626)	June 2009	Chooj, The road of Bandar Abbas to Minab, Hormozgan province	Leaves, Flowers	20
18	*Taverniera spartea *DC. (IMPH 627)	June 2009	The road of Bandar Abbas to Minab, Hormozgan province	Stem	26
19	*Tephrosia persica *Boiss. (IMPH 628)	June 2009	The road of Bandar Abbas to Minab, Hormozgan province	Aerial parts	19
20	*Trifolium campestre *Schreb. (IMPH 629)	May 2009	Golshan, Bandar Anzali, Gilan province	Leaves, Flowers	13.1
21	*Trifolium repens *L. (IMPH 630)	May 2009	Galangoodeh, Bandar Anzali, Gilan province	Aerial parts	21
22	*Trigonella spruneriana *Boiss. (IMPH 631)	May 2009	The road of Quhin to Loshan, Qazvin province	Aerial parts	10
23	*Vicia peregrina *L. var. *peregrine *(IMPH 632)	May 2009	The road of Quhin to Loshan, Qazvin province	Aerial parts	23.5

.^a^ Institute of Medicinal Plants Herbarium

*Preparation of the crude extracts and fractions*

The shade-dried powdered plant samples (20 g) were extracted with methanol in soxhlet apparatus for 12 h. The methanol extracts were concentrated using rotary vacuum distillation below 50°C under reduced pressure to get the crude extracts (Table 1) (5).

Among the screened extracts, anyone showing potent activity against the brine shrimp was resuspended in water and partitioned with chloroform (CHCl_3_) to separate less polar, water-insoluble compounds (5).

*Toxicity testing against the brine shrimp*

*Hatching shrimp*

Brine shrimp was obtained from Salt Creek, Inc. (Salt Lake City, UT 84104, USA). Brine shrimp eggs, *Artemia salina *Leach, were hatched in artificial seawater prepared through dissolving 38 g of sea salt in 1 L of distilled water. After 48 h of incubation at room temperature (27-29°C), the larvae (nauplii) were attracted to one side of the vessel with a light source and collected with pipette. Nauplii were separated from eggs through being aliquoted three times in small beakers containing seawater (6).

*Brine shrimp assay*

The bioactivity of the extracts was monitored with the brine shrimp lethality assay (7); 50 mg of methanol extracts were exactly measured and dissolved in 10 mL of DMSO to get a concentration of 5 mg/mL. From the stock solutions, different volumes were placed in 22 different vials making the volume up to 5 mL by the NaCl solution. The final concentration of the samples in the vials became 0.1, 0.2, 0.3, 0.4, 0.5, 1, 2, 5, to 140 μg/mL (ppm), respectively. Serial dilutions were made in triplicate (8).

Ten brine shrimp nauplii were then placed in each vial. Both positive (thymol) (9, 10) and negative (seawater containing DMSO) control assays were carried out in order to verify the susceptibility of *Artemia *under the assay conditions employed. For the negative control test of each vial, one vial containing the same volume of DMSO plus seawater up to 5 mL was used. After 24 h of incubation, the vials were observed using a magnifying glass and the numbers of survivors in each vial were counted and noted (8). In cases where control deaths occurred, the data was corrected using Abbott’s formula (%deaths = [(test – control)/ control] × 100) described by Rasoanaivo and Ratsimamanga-Urverg (11). The LC_50_ values were determined from the 24 h counts. In cases where data were insufficient for this technique, the dose-response data were transformed into a straight line by means of a logit transformation; the LC_50 _values were derived from the best-fit line obtained by linear regression analysis (7). The extract or isolated compounds were considered bioactive when LC_50_ value was lower than 30 μg/mL (6).

*Radical scavenging activity using DPPH method*

The DPPH radical-scavenging activity of the nine plants with LC_50_ value lower than 40 μg/mL was determined using the method proposed by Afolayan *et al*. A solution of 0.135 mM DPPH in methanol was prepared and 1.5 mL of this solution was mixed with 1.5 mL of the extract in methanol containing 2-1000 μg/mL concentration of the extract. The reaction mixture was vortexed completely and left in the dark at room temperature for 30 min. The absorbance of the mixture was measured through spectrophotometry at the 517 nm. These tests were carried out in triplicate and ascorbic acid was used as the reference (12).

Lower absorbance of the reaction mixture indicates higher DPPH° scavenging activity. DPPH° scavenging activity was calculated using the following formula: DPPH° scavenging activity (%) = [1 – (S – SB)/(C – CB)] × 100%, where S, SB, C and CB were the absorbances of the sample, the blank sample (1.5 mL of methanol plus 1.5 mL of sample at different concentrations), the control (1.5 mL of DPPH° solution plus 1.5 mL of methanol), and the blank control (methanol), respectively (13).

A percent inhibition versus the concentration curve was plotted and the concentration of the sample required for 50% inhibition was determined and expressed as IC_50 _value (14).

*Statistical analysis*

All the experimental results were as mean ± SD of three parallel measurements. The data was entered into a Microsoft Excel^©^ database and analyzed using SPSS (version 15.0). The LC_50_ and IC_50_ values were obtained by the linear regression analysis. Extracts giving LC_50_ values lower than 30 μg/mL were considered to be cytotoxic. The extracts with IC_50_ values lower than 50 μg/mL showed antioxidant activity.

## Results and Discussion

*Yield of plant extracts*

The percent yield of crude extracts following the removal of solvent using a rotary evaporator, were 10% for *Crotalaria burhia *and *Trigonella spruneriana *to 34% for *Glycyrrhiza glabra *var. *glabra *(Table 1).

*Cytotoxicity of plant extracts*

Results of the toxicity of the extracts against brine shrimp (LC_50_ values) are shown in Table 2. A total of 23 methanol extracts were tested for their toxicity against the brine shrimp using the brine shrimp lethality assay. The extracts of *Taverniera spartea *and *Tephrosia persica *showed significant cytotoxicity against brine shrimp (LC_50_ < 30 μg/mL) with LC_50_ values of 0.34 and 2.43 μg/mL, respectively, whereas the positive control, thymol, showed a LC_50_ value of 1.37 μg/mL. Chloroform fraction of these two species (*Taverniera spartea *and *Tephrosia persica*) represented different cytotoxicity against the brine shrimp with LC_50_ values of 113.79 and 1.23 μg/mL, respectively. These results suggested that the total extract of *Taverniera spartea *was more cytotoxic than its less polar fraction. But in the case of *Tephrosia persica*, chloroform fraction had cytotoxic effect as well as total methanol extract (Table 2).

**Table 2:** Brine shrimp lethality assay results of extracts or fractions of 23 plant species of the Leguminosae family

**No.**	**Plant species**	**Solvent**	**LC**_50_ **value (μg/mL)**^a^
1	*Astragalus squarrosus*	Methanol	54.91 ± 0.38
2	*Caesalpinia gilliesii*	Methanol	36.67 ± 0.59
3	*Crotalaria burhia*	Methanol	44.45 ± 0.39
4	*Gleditschia caspica*	Methanol	37.10 ± 0.16
5	*Glycyrrhiza glabra *var. *glabra*	Methanol	50.41 ± 0.73
6	*Glycyrrhiza glabra *var. *glandulifera*	Methanol	44.51 ± 0.48
7	*Indigofera articulata*	Methanol	42.43 ± 0.23
8	*Indigofera intricata*	Methanol	44.08 ± 0.45
9	*Lathyrus annus*	Methanol	> 90
10	*Lathyrus sativus *var. *stenophyllus*	Methanol	45.42 ± 0.31
11	*Lotus corniculatus *subsp. *corniculatus*	Methanol	32.00 ± 0.14
12	*Medicago rigidula*	Methanol	35.48 ± 0.17
13	*Melilotus indicus*	Methanol	72.52 ± 0.80
14	*Onobrychis altissima*	Methanol	51.38 ± 0.89
15	*Sophora alopecuroides*	Methanol	73.11 ± 1.20
16	*Sophora pachycarpa*	Methanol	56.73 ± 0.55
17	*Taverniera cuneifolia*	Methanol	39.39 ± 0.55
18	*Taverniera spartea*	Methanol	0.34 ± 0.01
19	*Taverniera spartea*	Chloroform	113.79 ± 1.43
20	*Tephrosia persica*	Methanol	2.43 ± 0.03
21	*Tephrosia persica*	Chloroform	1.23 ± 0.03
22	*Trifolium campestre*	Methanol	32.97 ± 0.17
23	*Trifolium repens*	Methanol	36.35 ± 0.59
24	*Trigonella spruneriana*	Methanol	52.41 ± 1.24
25	*Vicia peregrina *var. *peregrina*	Methanol	92.58 ± 1.07
26	Thymol^b^	-	1.37 ± 0.005

^a^ All values are the means of three measurements ± SD. ^b^Positive control

The extracts of twelve species including *Caesalpinia gilliesii*, *Crotalaria burhia*, *Gleditschia caspica*, *Glycyrrhiza glabra *var. *glandulifera*, *Indigofera articulata*, *Indigofera intricata*, *Lathyrus sativus *var. *stenophyllus*, *Lotus corniculatus *subsp. *Corniculatus*, *Medicago rigidula*, *Taverniera cuneifolia*, *Trifolium campestre *and *Trifolium repens *presented moderate cytotoxicity (LC_50_ between 30 and 50 μg/mL) against the brine shrimp. The extracts of *Lathyrus annus *and *Vicia peregrina *var. *peregrina *did not show any significant cytotoxicity (LC_50 _> 90 μg/mL) (Table 2). Since in most cases the toxicity is associated with pharmacological properties, it was deduced that the extracts of *Taverniera spartea *and *Tephrosia persica *had the best bioactivity.

*Antioxidant activity of the plant extracts*

IC_50_ values of DPPH percent scavenging activity of extracts and LC_50 _value of brine shrimp assay are given in Table 3, as calculated from the percent inhibition versus the concentration of extract curves. *Gleditschia caspica *and *Taverniera spartea *showed significant antioxidant activity (IC_50_ < 50 μg/mL) with IC_50_ values of 14.54 and 20.32 μg/mL, respectively, whereas the positive control, ascorbic acid, showed an IC_50_ value of 8.22 μg/mL. Five species including *Taverniera cuneifolia*, *Lotus corniculatus *subsp. *corniculatus*, *Trifolium campestre*, *Tephrosia persica *and *Trifolium repens *presented moderate antioxidant activity (IC_50_ between 50 and 200 μg/mL). Two species including *Caesalpinia gilliesii *and *Medicago rigidula *represented the highest IC_50 _value (205.41 and 423.13 μg/mL, respectively).

**Table 3:** IC_50_ values related to the DPPH assays of extracts with brine shrimp lethality assay LC_50_ value less than 40 μg/mL

**No.**	**Plant species**	**Brine shrimp assay LC**_50_ **(μg/mL)**^a^	**DPPH assay IC**_50_ **(μg/mL)**^a^
1	*Medicago rigidula*	35.48 ± 0.17	423.13 ± 0.05
2	*Caesalpinia gilliesii*	36.67 ± 0.59	205.41 ± 0.04
3	*Trifolium repens*	36.35 ± 0.59	180.78 ± 0.13
4	*Tephrosia persica*	2.43 ± 0.03	117.46 ± 0.09
5	*Trifolium campestre*	32.97 ± 0.17	71.88 ± 0.04
6	*Lotus corniculatus *subsp. *corniculatus*	32.00 ± 0.14	70.95 ± 0.01
7	*Taverniera cuneifolia*	39.39 ± 0.55	56.29 ± 0.02
8	*Taverniera spartea*	0.34 ± 0.01	20.32 ± 0.01
9	*Gleditschia caspica*	37.10 ± 0.16	14.54 ± 0.01
10	Thymol^b^	1.37 ± 0.005	-
11	Ascorbic acid^b^	-	8.22 ± 0.001

^a^ All values are the means of three measurements ± SD. ^b^Positive control

Cancer is a big challenge in the world as the suitable remedy is very expensive and even impossible in some cases. Many scientists are now engaged to find a potent remedy for cancer through the discovery of new and effective chemotherapeutic agents from plants and other sources (8).

A number of studies have reported the antioxidant or cytotoxic activity of some medicinal plants. For instance, 16 selected plants, which were collected from different localities of Yemen, have been evaluated for antimicrobial, antioxidant and cytotoxic activity and phytochemical screening (15). In another study, the cytotoxic potential of the different solvent extracts of the *Sapium baccatum *leaves, six column fractions of petroleum ether extract and three pure compounds have been determined by using brine shrimp lethality assay. The LC_50_ of all the tested samples were showed to be lethal to brine shrimp nauplii (16). Radical scavenging activity of the essential oils of *Zataria multiflora *from different parts of Iran has been determined. In the DPPH antioxidant assay, all samples exhibited a remarkable activity (IC_50_ = 19.7 μg/mL) almost similar to BHT (IC_50_ = 18.1 μg/mL) (17). Bioassay screening of the essential oil and various extracts of *Heracleum persicum *fruits and rhizomes of *Zingiber officinale *have been studied using brine shrimp cytotoxicity assay (18). The extracts of *Alnus glutinosa*, *Fraxinus excelsior *and *Papaver rhoeas *have been screened for their antioxidant and antibacterial activity, as well as their general toxicity towards brine shrimps (19).

Brine shrimp lethality assay is a primary assay to detect cytotoxic property of plant extract and, further studies are required to establish the cytotoxicity of the plant extracts against human cancer cell lines. However, our results in this study may predict that which species of Leguminosae family will give better results on cancer cell lines. Although the crude extract or chloroform fraction have been examined in the present study, further investigations using single components from these extracts may explore potent cytotoxic properties.

This is the first report on cytotoxicity screening of these twenty-three plant species of Leguminosae family from different regions of Iran. However, according to the criteria of the American National Cancer Institute, the LC_50_ limit to consider a crude extract promising for further purification is lower than 30 μg/mL (20). Thus, only two species among 23 tested species of plants presented significant cytotoxicity against brine shrimp. The extracts of *Taverniera spartea *and *Tephrosia persica *could be considered as the potential sources of anticancer compounds.

Another mechanism of cancer prevention might be the radical scavenging of free radical oxygen and other species associated with cancer cell development. However, the results of radical scavenging activity with DPPH showed that two samples out the 9 tested samples were more active (*Gleditschia caspica *and *Taverniera spartea*; IC_50_ < 50 μg/mL).

Among the 23 tested plant species, *Taverniera spartea *had the most cytotoxic and antioxidant activity and was the best candidate for these effects. Further investigations are necessary for the chemical characterization of the active compounds and more comprehensive biological assays.
